# Comparison of Thermal Imaging and Magnetic Resonance Imaging in Evaluating the Extension of Diabetic Foot Infections: A Report of Two Cases

**DOI:** 10.7759/cureus.81942

**Published:** 2025-04-09

**Authors:** Abdul Rahim Mohamed Sirajudeen, Chooi Leng Low, Mohd Radhwan Abidin, Muhammad Wafiuddin Ahmad, Ahmad Syahrizan Sulaiman, Ahmad Hafiz Zulkifly, Ren Yi Kow

**Affiliations:** 1 Department of Orthopaedics, Traumatology and Rehabilitation, International Islamic University Malaysia, Kuantan, MYS; 2 Department of Radiology, International Islamic University Malaysia, Kuantan, MYS; 3 Research Excellence Management Centre, Universiti Malaysia Pahang Al-Sultan Abdullah, Pekan, MYS

**Keywords:** diabetic foot evaluation, diabetic foot infection, diabetic foot ulceration, magnetic resonance (mr), thermal imaging camera

## Abstract

Diabetic foot disease is a severe complication of diabetes mellitus that can lead to infection, ulceration, and amputation. Accurate assessment of the extent of infection is crucial for effective treatment. Advancements in technology, such as thermal imaging, have shown promise as bedside adjuncts in diabetic foot assessment. This report of two cases compares the efficacy of thermal imaging and the gold-standard magnetic resonance imaging (MRI) in evaluating the extent of diabetic foot infections in two patients. Thermal imaging and MRI were used preoperatively to assess diabetic foot infections. Thermal imaging showed a reduced temperature around the ulcer, correlating with necrotic tissues; however, it lacked the comprehensive detail provided by MRI. MRI accurately delineated the depth and extent of infection, identified potential bone involvement, and detected subtle soft tissue changes, features that thermal imaging lacked. MRI remains indispensable for its superior ability to provide detailed anatomical information and assess the full extent of infection, which is critical for surgical decision-making and long-term management. The complementary use of both modalities may enhance diagnostic accuracy, with thermal imaging serving as an accessible preliminary tool and MRI providing the necessary depth for complex cases.

## Introduction

Diabetes mellitus is a major non-communicable disease, with a rising prevalence contributing to significant morbidity and healthcare burden [1-7]. It is associated with a wide range of macrovascular and microvascular complications, including cardiovascular disease, nephropathy, neuropathy, and diabetic foot disease [1-3]. Among these complications, diabetic foot ulcers (DFUs) are particularly concerning, affecting up to 15% of diabetic patients over their disease course [4,5]. Diabetic foot infection (DFI) is a serious complication that arises from a complex interplay of peripheral neuropathy, ischemia, and persistent ulceration, often leading to severe morbidity and increased healthcare costs [1-3].

One of the primary challenges in managing DFI is accurately determining the extent of infection, which is crucial for guiding appropriate treatment strategies [8]. The severity and depth of infection influence the choice of medical therapy, the need for surgical intervention, and the level of amputation required [1]. In cases where the infection extends proximally, higher-level amputations may be necessary to prevent further systemic spread [5,6]. Traditional methods for assessing the extent of infection include clinical examination, biochemical markers of inflammation, and radiographic imaging. However, these approaches have limitations in sensitivity and specificity, necessitating more advanced imaging techniques.

Magnetic resonance imaging (MRI) is currently regarded as the gold standard for evaluating the depth and extent of infection in patients with DFI. MRI provides superior soft tissue contrast, enabling precise visualization of deep infections, abscess formation, and osteomyelitis. However, despite its diagnostic accuracy, MRI is not routinely performed in all cases due to high cost, limited availability, and potential delays in definitive surgical management. As a result, there is growing interest in alternative imaging modalities that can provide rapid, cost-effective, and accessible diagnostic support for clinicians managing DFI.

Infrared thermography (thermal imaging) has been explored as a potential adjunct in the assessment of DFIs [9,10]. It is a non-invasive, radiation-free technique that detects variations in skin temperature, which may correlate with inflammatory changes, infection, and impaired tissue perfusion. Previous studies have suggested that thermal imaging can aid in the early detection of DFUs, assess infection severity, and monitor treatment response [9,10]. However, there is limited research directly comparing the accuracy of thermal imaging with MRI in assessing the extent of infection in DFI patients. Here, we compare the efficacy of thermal imaging and MRI in assessing the extension of the DFI in two patients. Both patients underwent thermal imaging and MRI preoperatively to assess the extent of infection. Thermal imaging was performed using a high-resolution infrared camera (FLIR C5 Thermal Camera), while MRI was performed using a 3.0 Tesla scanner.

## Case presentation

Case 1

**Figure 1:**
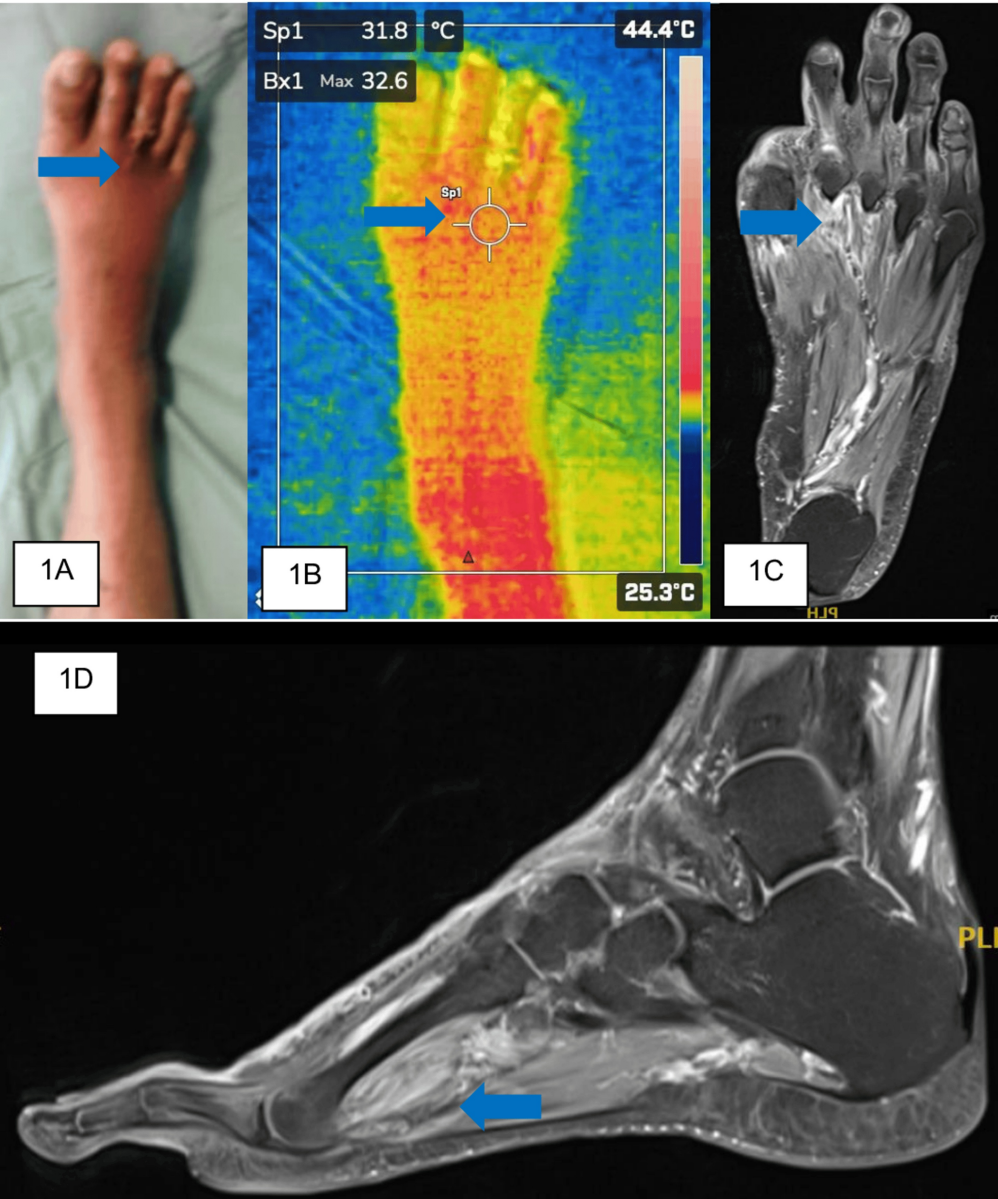
(A) Clinical photo of the affected foot with the area of interest marked by the blue arrow. (B) Thermal imaging of the affected foot upon admission to the ward. (C) Axial view of the T1 post-contrast MRI image with the affected area (arrow). (D) Sagittal view of the T1 post-contrast MRI image clearly delineating the area of interest (blue arrow).

A 59-year-old male with a 15-year history of type 2 diabetes mellitus presented with pain and swelling over the right third toe for two weeks, associated with blackish discoloration of the affected toe one week before presentation. The patient reported no fever or history of trauma and maintained independent ambulation. Examination of the foot revealed erythema extending to the midfoot level, with an ulcer over the third interdigital webspace accompanied by serous discharge, and a blackish discoloration of the third toe (Figure 1A). Distal pulses were intact, with reduced sensation over the stocking distribution. Blood investigations showed raised septic parameters such as C-reactive protein (CRP) and white blood cell count (WBC) with mild acute kidney injury. Radiography of the foot was unremarkable, showing no osteomyelitis changes over the third toe or presence of gas shadows. Thermal imaging showing a reduced temperature at the site of the ulcer (31.8°C compared to an average of 32.6°C over other parts of the foot) with extension just proximal to the metatarsophalangeal joint (Figure 1B). MRI confirmed the presence of extensive soft tissue involvement beyond the region of the insult, with the edema more prominent around the third toe. MRI of the right foot did not reveal signs of focal collection but detected hyperintense signal on T1 (Figure 1C), hypointense signals on turbo inversion recovery magnitude with contrast enhancement post-gadolinium extending from the subcutaneous layer of the third toe to the mid-foot (Figure 1D).

The patient was diagnosed with right third toe wet gangrene and underwent ray amputation of the right third toe. Intraoperatively, only a small area of unhealthy tissue was observed proximal to the amputation site, along with wet gangrene of the third toe. Tissue culture and sensitivity analysis identified *Staphylococcus aureus* as the infective organism, which demonstrated sensitivity to cloxacillin. The patient received antibiotic therapy for six weeks and was assessed during serial follow-up appointments. The patient was discharged with a well-healed wound over the operative site at six weeks postoperatively.

Case 2

**Figure 2:**
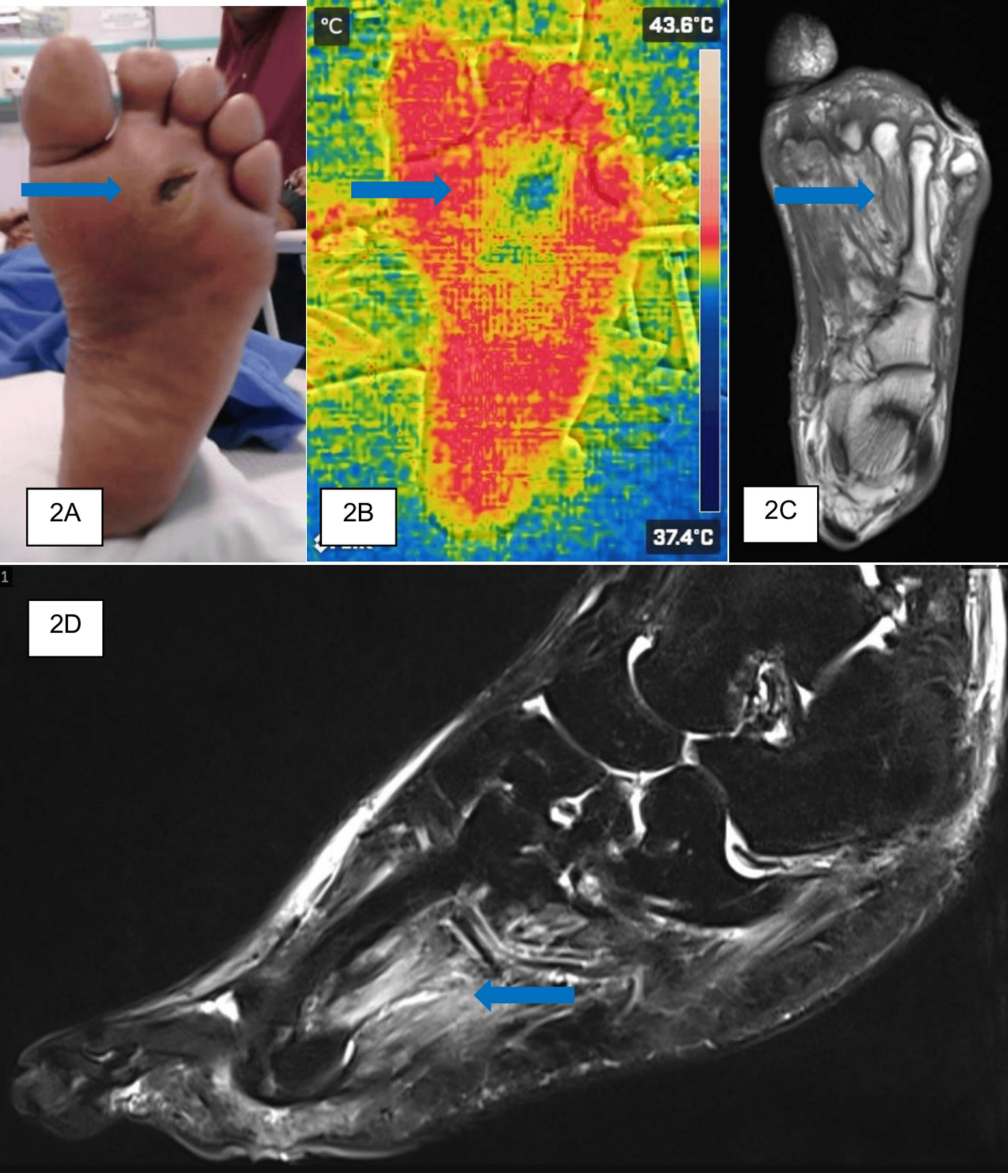
(A) Clinical figure of the affected foot, with the area of interest marked with the blue arrow. (B) Thermal imaging of the affected foot upon admission to the ward. (C) Axial view of the T1 MRI image of the affected foot. (D) Sagittal view of the T2 fat-saturation MRI image of the affected foot, with the border and depth of the infection clearly demarcated in the MRI image.

A 72-year-old male with a 30-year history of type 2 diabetes mellitus presented with a deep ulcer on the plantar aspect of his left foot. The ulcer had been present for one week, accompanied by edema, erythema, and increased local temperature. Upon examination, an ulcer measuring 2 cm × 2 cm with purulent discharge and surrounding callosity was observed (Figure 2A). Distal pulses were palpable, with elevated septic parameters such as CRP and WBC. There was also mild acute kidney injury. Radiography of the foot did not reveal osteomyelitis changes or gas shadows. Thermal imaging showed temperature reduction around the ulcer (40.1°C compared to 42°C over the surrounding area) (Figure 2B). MRI of the left foot did not detect any focal collection on the coronal view (Figure 2C), but noted extension of subcutaneous edema over the plantar surface of the third and fourth proximal phalanx extending up to the heel on the T2 sagittal view (Figure 2D).

The patient was diagnosed with an infected left DFU Wagner 3 and underwent wound debridement of the left foot. Intraoperative findings included an ulcer over the plantar aspect of the foot measuring 2 × 3 cm with compromised underlying tissue approximately 1 cm proximal to the wound. Culture and sensitivity analysis demonstrated mixed growth, and the patient received antibiotic therapy for six weeks. The wound demonstrated satisfactory healing at six weeks postoperatively.

## Discussion

One of the challenges in the management of DFI is accurately determining the extent of the infection, which is crucial for appropriate treatment planning [8]. The extent of infection influences clinical decisions, such as the level of amputation required. Traditionally, clinical palpation, biochemical investigations, and radio-imaging assessments, including MRI, are employed with caution to assess infection depth and spread. Despite MRI being the gold standard for detailed delineation of infection in DFI, its high cost and potential to delay definitive surgical management limit its frequent use. Infrared thermography has found applications in various medical fields, such as diagnosing odontogenic facial cellulitis and detecting COVID-19 infections in patients with minimal symptoms [11,12]. More recently, its role in assessing vascularity in peripheral artery disease and monitoring post-surgical infection sites has been explored [13]. Infrared thermography has also been utilized in orthopedic settings to detect pin-site infection [14].

Despite the early promising results, our findings in these two cases indicate that thermal imaging has limited value in assessing acute DFIs when compared to MRI. Thermal imaging showed reduced temperature around the ulcer, which likely resulted from necrotic tissues that did not exhibit raised temperatures. This observation correlates with the study by Ilo et al., which demonstrated that the local temperature of high-risk diabetic foot is higher than that of a normal foot, as well as the findings of Rahbek et al. on digital thermography for infection monitoring post-surgery [13,14]. Contrary to these studies, Hutting et al. suggested that infrared thermography is not valuable for monitoring DFIs during in-hospital treatment, which we concur with our findings in these two cases [10].

The thermal imaging only indicated reduced temperatures over the foci of infection, which does not provide sufficient information on the extent of infection necessary for surgical planning. MRI, on the other hand, accurately delineated the depth and extent of infection and identified potential bone involvement and subtle soft tissue changes. The MRI findings in our cases revealed extensive edema and precise anatomical details, which were crucial for comprehensive evaluation and effective surgical planning. In contrast, the thermal imaging showed an ill-defined temperature spike surrounding the infective area, indicating the edema and inflammation secondary to the infection, but lacked the accurate delineation needed for surgical planning. 

Given the limitations of thermal imaging observed in these two cases, it is not a reliable tool for assessing acute DFIs. In the initial planning, we planned to evaluate a total of 10 patients as part of the pilot study to compare the efficacy of thermal imaging and MRI in evaluating the extension of DFI. The recruitment for this study was terminated after reviewing the provisional results of these two cases, as the thermal imaging did not demonstrate the necessary capability to aid in surgical planning and infection assessment.

In summary, while thermal imaging offers a non-invasive, cost-effective, and rapid screening option for initial assessment, it is insufficient for detailed evaluation of DFIs. MRI remains indispensable for its superior ability to provide detailed anatomical information and assess the full extent of infection, which is critical for surgical decision-making and long-term management strategies. The complementary use of both modalities may enhance diagnostic accuracy, with thermal imaging serving as an accessible preliminary tool and MRI providing the depth required for complex cases. This combined approach could improve patient outcomes by informing more precise treatment plans and potentially reducing the need for extensive surgeries.

## Conclusions

The two cases reported here demonstrate that while infrared thermography offers a non-invasive, cost-effective, and rapid screening option for initial assessment of DFIs, it is insufficient for detailed evaluation and surgical planning. MRI remains an indispensable modality for its superior ability to provide detailed anatomical information and assess the full extent of infection. The complementary use of both modalities may enhance diagnostic accuracy, with thermal imaging serving as an accessible preliminary tool and MRI providing the depth required for complex cases. Future research should focus on exploring the integration of advanced imaging technologies and machine learning algorithms to enhance the diagnostic capabilities of thermal imaging. Additionally, large-scale studies are needed to validate these findings and assess the potential of combined imaging approaches in improving patient outcomes in DFI management.

## References

[1] Kow RY, Low CL, Ruben JK, Zaharul-Azri MZ, Lim BC (2019). Predictive factors of major lower extremity amputations in diabetic foot infections: a cross-sectional study at District Hospital in Malaysia. Malays Orthop J.

[2] Alsaigh SH, Alzaghran RH, Alahmari DA, Hameed LN, Alfurayh KM, Alaql KB (2022). Knowledge, awareness, and practice related to diabetic foot ulcer among healthcare workers and diabetic patients and their relatives in Saudi Arabia: a cross-sectional study. Cureus.

[3] Ahmad S, Khan MS, Shah MH, Khan A, Bano R, Qazi M (2022). Microbial profile and antimicrobial susceptibility pattern in diabetic foot ulcer patients attending a tertiary care hospital. Cureus.

[4] Elghoneimy YA, Alkabah AA, Alalsayedsalih HM (2022). Risk factors and surgical outcomes of diabetic foot in diabetic patients at King Fahad University Hospital. Cureus.

[5] Alotaibi A, Alqhtani N, Alluhaymid A (2024). Awareness of diabetic patients in the Qassim region about diabetic foot and its complications. Cureus.

[6] Kow RY, Low CL, Ruben JK, Zaharul Azri WM, Mor Japar Khan ES (2019). Microbiology of diabetic foot infections in three district hospital in Malaysia and comparison with South East Asian Countries. Med J Malaysia.

[7] Kow RY, Low CL, Ayeop M, Che-Ahmad A, Awang MS (2022). Characteristics and microbiological profile of patients with diabetic foot infections in Kuantan, Pahang. Malays Orthop J.

[8] Behera KK, Soren UK, Behera BK, Devi S (2024). Studying the diabetic foot at risk using a 60-second foot screening tool and the importance of the categories of the foot at risk in diabetes patients at a tertiary care center in East India. Cureus.

[9] Zakaria SA, Low CL, Kow RY (2024). Thermography research in diabetic foot: insights from a Scopus-based bibliometric study. Cureus.

[10] Hutting KH, Aan de Stegge WB, Kruse RR, van Baal JG, Bus SA, van Netten JJ (2020). Infrared thermography for monitoring severity and treatment of diabetic foot infections. Vasc Biol.

[11] Derruau S, Bogard F, Exartier-Menard G, Mauprivez C, Polidori G (2021). Medical infrared thermography in odontogenic facial cellulitis as a clinical decision support tool. A technical note. Diagnostics (Basel).

[12] Martinez-Jimenez MA, Loza-Gonzalez VM, Kolosovas-Machuca ES, Yanes-Lane ME, Ramirez-GarciaLuna AS, Ramirez-GarciaLuna JL (2021). Diagnostic accuracy of infrared thermal imaging for detecting COVID-19 infection in minimally symptomatic patients. Eur J Clin Invest.

[13] Ilo A, Romsi P, Mäkelä J (2020). Infrared thermography and vascular disorders in diabetic feet. J Diabetes Sci Technol.

[14] Rahbek O, Husum HC, Fridberg M, Ghaffari A, Kold S (2021). Intrarater reliability of digital thermography in detecting pin site infection: a proof of concept study. Strategies Trauma Limb Reconstr.

